# Highly Permeable Mixed Matrix Hollow Fiber Membrane as a Latent Route for Hydrogen Purification from Hydrocarbons/Carbon Dioxide

**DOI:** 10.3390/membranes11110865

**Published:** 2021-11-10

**Authors:** Yu-Ting Lin, Ming-Yen Wey, Hui-Hsin Tseng

**Affiliations:** Department of Environmental Engineering, National Chung Hsing University, Taichung 402, Taiwan; a0938866458@gmail.com

**Keywords:** hollow fiber, mixed matrix membrane, alumina powder, gas separation

## Abstract

This work reported on the fabrication and investigation of a mixed matrix hollow fiber membrane (MMHFM) by incorporating commercially available alumina particles into a polyetherimide (PEI) polymer matrix. These MMHFMs were prepared by the dry-wet spinning technique. Accordingly, optimizing the spinning parameters, including the air gap distance and flow rate ratio, is key to determining the gas separation performance. However, there are few studies regarding the effect of the filler dimensions. Consequently, three sizes of alumina particles, 20 nm, 30 nm, and 1000 nm, were respectively added into the PEI phase to examine the influence of filler size on gas permeation property. Moreover, the permeation properties of lower hydrocarbons (i.e., ethane and propane) were also measured to evaluate potential for emerging applications. The results indicated the as-synthesized membrane exhibited a remarkable hydrogen permeance of 1065.24 GPU, and relatively high separation factors of 4.53, 5.77, and 5.39 for H_2_/CO_2_, H_2_/C_2_H_6_, and H_2_/C_3_H_8_, respectively. This resulted from good compatibility between the larger fillers and the PEI polymer, as well as a reduction in the finger-like voids. Overall, the MMHFM in this work was deemed to be a promising candidate to separate hydrogen from gas streams, based on the comparison of the separation performance against other reported studies.

## 1. Introduction

Undoubtedly, faced with the threats of depletion of petrochemical resource, catastrophic extreme weather, and burgeoning energy demand, all countries are driven to seek for renewable resources for sustainable development [1]. Among a variety of sustainable resources known to date, hydrogen is a compelling one, because of its high heating value and zero-carbon emission. However, there exists a gap of about 10–40% in the hydrogen purity between production and application [2]. Therefore, the purification process is necessary to achieve a hydrogen-based economy, and therein, the membrane-based separation process is considered as a viable option to fulfill the goal of desirable hydrogen purity, owing to merits such as energy-saving and operation flexibility [3].

The commonly used membranes for hydrogen purification contain metal membrane, carbon membrane, and polyimide/polysulfone-derived polymeric membrane [2]. Based on the used materials, hydrogen-separation membrane could be classified into two types, namely inorganic membrane and organic membrane. Both membrane types have their pros and cons. Conventionally, inorganic membranes exhibit good thermal/chemical stability and are considered a promising separation technology, although their commercial application is hampered by their complex fabrication process and high production cost [4]. On the other hand, polymeric membranes are vulnerable to corrosion in demanding environments, despite their straightforward manufacturing process. In addition, from industrial perspectives, the hydrogen capacity of widely used membranes, regardless of used material, are still insufficient, as the hydrogen production rate in a Japanese plant reached up to 50 N m^3^/hour [5]. Hence, mixed matrix membranes (MMM), which are prepared by incorporating an inorganic filler into the polymer matrix, are the preferred solution in academic and industry fields to overcome the above-mentioned limitations, via a synergistic interaction between the organic and inorganic components [6,7]. Moreover, compared with flat-sheet and tubular membranes, hollow fiber configurations have an important place in a broad range of applications. Given their exceptionally high surface area per volume, hollow fiber configurations enable membrane units to possess a high packing density and offer superior separation efficiency, and have, therefore, received increased attention from researchers. For this reason, it is desirable to further develop the mixed matrix hollow fiber membrane (MMHFM) to improve membrane durability, simplify the fabrication stage, and increase the membrane separation efficiency.

Nevertheless, the problems of MMHFMs are the interfacial interactions between the polymer matrix and inorganic fillers, commonly resulting in the agglomeration of the filler and inferior membrane performance. Inferior polymer–inorganic filler interactions, such as particle agglomeration, pore blockage, void formation, and polymer rigidification, not only lower the gas separation performance, but also deteriorate the physical properties of mixed matrix membranes [8].

Laghaei et al. have argued that surface-modified mesoporous silica facilitates better interactions with the polymer [9]. Along similar lines, Xing et al. [10] have argued that functionalized silica microspheres mixed with sulfonated polyether ether ketone (SPEEK) and used to fabricate MMMs exhibit a ${\mathrm{CO}}_{2}$ permeability increase of 160% to 310% compared to neat SPEEK membrane, due to the strong interaction between the fillers and polymer. Dai et al. [11] indicated that the incorporation of functionalized graphene oxide in poly(ether-b-amide) (PEBAX) results in fabricated MMMs with a high selectivity of ${\mathrm{CO}}_{2}$ separation, because of the graphene materials. Based on the published literature, it should be noted that screening the suitable fillers and corresponding polymer matrix plays a vital role in determining the separation performance of as-fabricated MMMs, because of the varying interface compatibility.

Consequently, the current study would focus on the influences of spinning parameters as well as particle size of nano-size fillers on the resultant MMHFM morphology and separation capability, in which three sizes of commercial alumina particles were selected as fillers, with polyetherimide (PEI) as the main matrix, because: (i) PEI has a moderate level of selectivity and great thermal stability to enable itself operated in the harsh condition; (ii) nano-size alumina particles can provide the selective surface flow characteristic, due to their mesoporous nature; (iii) the synergism effect could be expected by the hydrogen bonding between both materials.

## 2. Experiment

### 2.1. Materials

The commercial polyetherimide (PEI, repeat unit molecular weight = 592 g/mol) was obtained from Sigma Aldrich (Saint Louis, MO, USA), and was utilized as the polymeric phase. The alumina (${\mathrm{Al}}_{2}O_{3}$) powder, with particle sizes of 1000, 30, and 20 nm as a dispersed phase, was purchased from Taiwan Union Abrasives Corp. (Kaohsiung City, Taiwan). The N-methyl-2-pyrrolidone (NMP) solvent was purchased from Macron Fine Chemicals Co., a flagship brand of Avantor Inc. (Radnor Township, PA, USA). The tested gases were supplied from Toyo Gas Co. (Taichung City, Taiwan), including H_2_, CO_2_, O_2_, N_2_, CO, C_2_H_6_, and C_3_H_8_. All reagents were used directly, without any purification.

### 2.2. Preparation of MMHFMs

Briefly, a phase inversion dry–wet spinning technique was employed to fabricate PEI-${\mathrm{Al}}_{2}O_{3}$ nanoparticle-based MMHFMs. First, the homogeneous PEI solution with a concentration of 25 wt. % was obtained by dissolving the PEI granules in the NMP solvent at a temperature of 80 °C and stirring rate of 60 rpm for one day. Once the polymer was completely dissolved, a certain amount of alumina powder with different particle sizes (1000, 30, and 20 nm) was added into the above polymer solution, to obtain a mixture with a fixed ${\mathrm{Al}}_{2}O_{3}$ loading weight (6 wt. %, based on the polymeric solution). The mixture was stirred for one day until it was homogeneous, and no agglomerates were observable. Then, the mixed solution was degassed overnight at room temperature, thus forming a spinning dope. The spinning dope was extruded through a tube-in-orifice spinneret (outer and inner diameters of 3.5 mm and 2.0 mm, respectively) into an external coagulant bath (tap water) using a pressurized inert gas (nitrogen) as the driving force to push the mixtures, producing nascent fibers. The air gap distance between the spinneret and external coagulant bath was set as 0 cm, 5 cm, and 10 cm, respectively. Upon reaching the bath, a coagulation of the fluid/solid mixture occurred, consolidating the hollow fiber structure. The lumen of the hollow fiber was formed by continuously injecting the bore liquid (deionized water) into the bore side of the spinneret throughout the spinning process. The flow rate ratio of dope to bore liquid was made equal to 5.14, 2.57, and 1.28 by changing the flow rate of the bore liquid and maintaining that of the dope. The parameters used in the spinning procedure are summarized in Table 1. Finally, the as-spun hollow fibers were left in the coagulation bath for 24 h to finish the coagulation process. Moreover, the resultant MMHFMs, with different parameters, were labeled as AGx-Ry-znm, where x refers to the adopted air gap distance, y refers to the value of the flow rate ratio, and z refers to the particle size of the incorporated alumina filler.

### 2.3. Characterizations

Morphological analyses for the produced MMHFMs were performed using field emission scanning electron microscopy (FE-SEM, JEOL JSM-6700F, JEOL Ltd., Tokyo city, Japan) at an accelerating voltage of 3.0 kV. Prior to SEM analysis, a thin platinum layer was coated onto the surface of each membrane sample to improve conductivity. Subsequently, the membrane thickness was measured on the basis of its cross-section image analysis. The distribution condition of the dispersive phase in the polymer matrix was characterized by the energy dispersive X-ray (EDX) spectroscopy mapping technique (Oxford Instruments, X-MAX80 Aztec operation system, Abingdon, Oxfordshire, UK). Thermogravimetric analyzer (TGA) was performed to examine the thermal stability of unfilled PEI HFM and optimal MMHFM under nitrogen atmosphere.

### 2.4. Permeation Experiment

The constant volume/variable pressure method [3] was applied to measure the pure gas permeation value through the obtained MMHFMs in this study using an in-house laboratory device, as described in our previous work [12], and an operation temperature of 28 ± 2 °C. Prior to permeation measurement, one end of the MMHFM was sealed by epoxy, forming a “dead-end” fiber. The dead-end fiber was then inserted into a stainless steel permeation rig, using epoxy to seal the space between the other side of fiber (open-ending) and stainless steel tube. It was then left to stand for a period of 24 h for the epoxy to fully cure, to prevent the potting spot from leaking. The permeation system was kept under vacuum using a vacuum pump to prevent atmospheric gas pollution, before feeding the gas at a constant pressure into the system. Finally, tested gas with a pressure of about 2 kg/cm^2^ was fed into the lumen side of the MMHFM mounted in the permeation module. The pressures of the feed side and downstream side of the permeation system were measured by digital pressure transducers (JPT-131S, Jetec Electronics Co., Ltd., Tokyo, Japan), and the detected data was recorded by computer. Therefore, the change in pressure at the downstream side over a period of time (*dp*/*dt*) was acquired, and we substituted this value into the following equation to calculate the corresponding permeance. The gas permeance was expressed as GPU:

(1) $$P=\left[\frac{dp}{dt}\right]\frac{V\cdot T_{0}}{A\cdot \Delta p\cdot T\cdot P_{0}}$$

where *P_g_* refers to the permeance of gas *g* (1 GPU = 10^−6^ × cm^3^ (STP)/cm^2^ · s·cmHg), *dp*/*dt* refers to the slope of the pressure change curve over time when the steady state is reached (cmHg/sec), V refers to the volume of the downstream side (cm^3^), Δ*P* refers to the difference in pressure of the gas between the feed side and permeate side of the membrane (cmHg), A refers to the effective area of the membrane (cm^2^), *P*_0_ and *T*_0_ refer to the pressure and temperature in the standard state (76 cmHg, 273 K), respectively, and T refers to the operation temperature (K).

The ideal selectivity for gas pair (α) was defined as the ratio of two gases in permeance and calculated according to Equation (2), as follows:

(2) $$\alpha =\frac{P_{i}}{P_{j}}$$

where *P*_i_ and *P*_j_ refer to the gas permeance of gas i and gas j, respectively (GPU).

## 3. Results and Discussion

### 3.1. The Distribution of Alumina Particles in the Resultant Membrane

**Figure 1:**
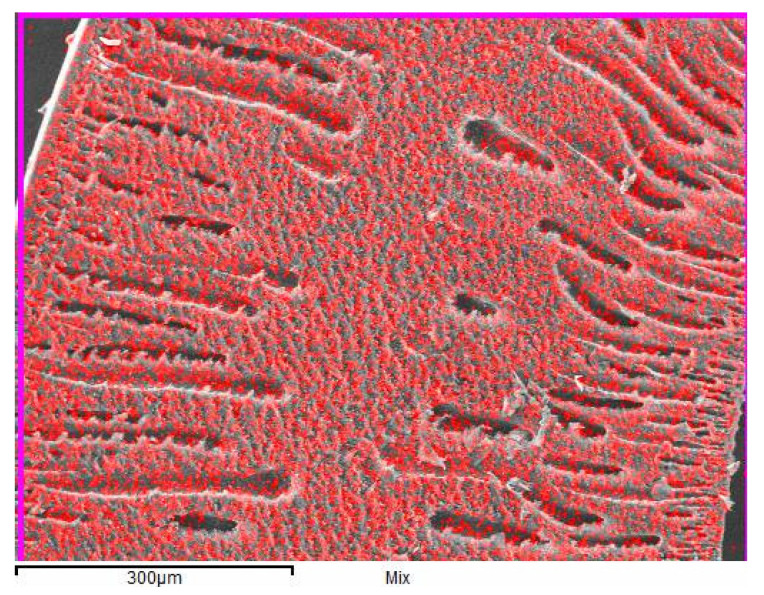
The EDX mapping analysis for the cross-sectional image of resultant MMHFM. The red dot represents the location of Al element.

Broadly, the gas separation performance of the resulting MMHFMs still suffered from the dispersion of inorganic fillers in the polymer matrix. Therefore, the spatial distribution of aluminum (Al) element in the cross-sectional images of the MMHFMs was detected by the EDX mapping technique to confirm the inorganic filler dispersion. The mapping result below (Figure 1) show the absence of agglomeration phenomena for alumina particles in the polymeric phase, thus verifying the dispersion of fillers is favorable. The improved dispersion could be attributable to the hydrogen bonding interactions between the hydroxyl group on the surface of the alumina particles with C-H segments in the PEI chains. Overall, the loading weight of inorganic fillers (6 wt. %) in this study was deemed suitable for adopting in the preparation of MMHFMs.

### 3.2. Morphological Structure Analysis

**Figure 2:**
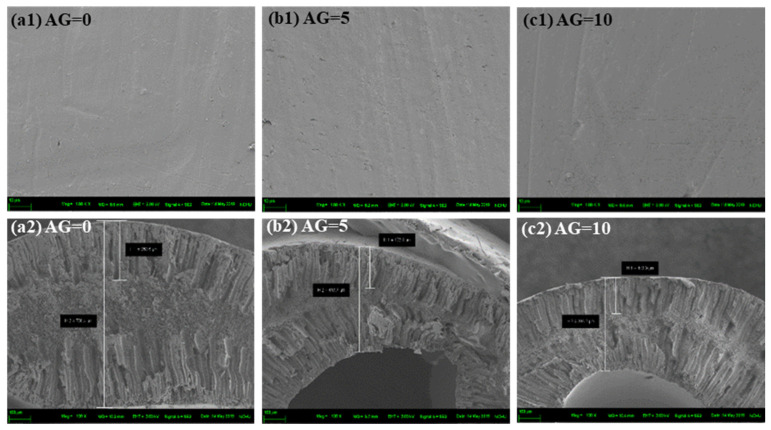
The outer-surface (**top**) and cross-section (**bottom**) SEM images of MMHFMs spun with different air gap distances: (**a1**,**a2**) AG0-R5.14-1000 nm, (**b1,b2**) AG5-R5.14-1000 nm, and (**c1**,**c2**) AG10-R5.14-1000 nm.

The outer surface and cross-sectional morphology images of the MMHFMs with varying air gap distances are displayed in Figure 2, and the corresponding membrane thicknesses are provided in Table 2. As can be seen from Figure 2a1–c1, when the air gap distance increased from 0 to 10 cm, the outer surface micrographs of the resultant MMHFMs remained smooth and defect-free. Moreover, all cross-sectional SEM micrographs, as shown in Figure 2a2–c2, exhibit an asymmetric membrane structure, where the finger-like voids occupied both the shell-side and bore-side of the hollow fiber with a sponge-like structure located in the middle of them, giving a sandwich-like structure. This structure is very commonly observed when the membrane preparation method is a non-solvent-induced phase separation process [13]. In addition, based on the cross-section images, the related membrane properties, including wall thickness of the membrane and the length of the finger-like region near the outer surface, are presented in Table 2. The membrane thickness of MMHFMs spun without an air gap (786.4 μm) was larger than that of the MMHFMs fabricated with a higherair gap distance, such as 5 and 10 cm. The die swell effect was triggered by virtue of the viscoelastic property of the polymer precursor solution, in the period of the polymeric macromolecule spun from the spinneret, thus causing a greater wall thickness of the hollow fiber. On the other hand, when a higherair gap distance was adopted, the influence of the die swell was offset by the elongation induced by the gravity force, reducing the enlargement of the hollow fiber. This means a larger air gap distance, resulting in a decrease in membrane thickness of the prepared membranes. This observed trend is in agreement with previously reported work [14]. We also observed the length of finger-like voids near the outer surface were shortened from 250.5 to 153.9 μm by increasing the air gap distance from 0 to 10 cm. A larger air gap distance will provide the nascent fibers a longer retention time to be exposed to the atmosphere environment. When the nascent fiber is exposed to the atmosphere for a longer period, the solvent on the outer surface of the fiber evaporates, which could be deemed as thermally induced phase separation to some extent, and subsequently, causes a delayed phase separation. The slower phase separation rate will lead to the formation of a dense sponge-like structure and suppression of finger-like voids. Similar results have been found in other studies [15,16].

**Figure 3:**
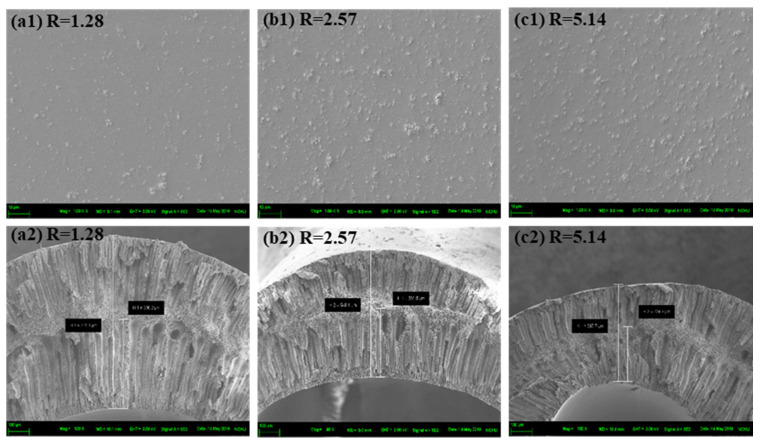
The inner-surface (**top**) and cross-section (**bottom**) SEM images of MMHFMs spun with different flow rate ratios: (**a1,a2**) AG10-R1.28-1000 nm, (**b1,b2**) AG10-R2.57-1000nm, and (**c1,c2**) AG10-R5.14-1000nm.

The flow rate ratio of the spinning solution to the bore liquid was then adjusted to investigate its effect on the morphology of the as-prepared MMHFMs (see Figure 3), while the air gap was held constant at 10 cm. Similarly, Table 3 presents the related membrane information with different flow rate ratios. All inter-surface micrographs of the developed MMHFMs prepared with different flow rate ratios are depicted in Figure 3a1–c1, showing the rough morphology; there were no manifest cracks or defects in the membrane surface. This suggests the fillers were perfectly wrapped in the polymer chains, and there were favorable interactions between the polymeric matrix and fillers [17], as evidenced in Figure 1. The typical sandwich-like structure is also displayed in Figure 3a2–c2. Furthermore, given the change in the flow rate ratio of the dope/bore fluid, the characteristics of the inner surface structure of the resulting MMHFMs changed. As reported in Table 3, when the flow rate ratio reduced from 5.14 to 1.28, the length of the finger-like pores originating from the inner surface increased from 224.7 μm to 366.2 μm. As the flow rate of the dope was fixed, the flow rate ratio presented here was inversely proportional to the flow rate of the bore solution. A low flow rate ratio will result in instant phase inversion and favor the formation of finger-like structures at the lumen side of hollow fiber. This is due to much more non-solvent (water) per unit time flowing into the lumen of the fiber [18,19]. Additionally, a higher flow rate of bore liquid will provide sufficient hydrodynamic force against the dope to obtain a thicker hollow fiber.

### 3.3. Single-Gas Permeance Test

#### 3.3.1. Effect of Air Gap Distance

**Figure 4:**
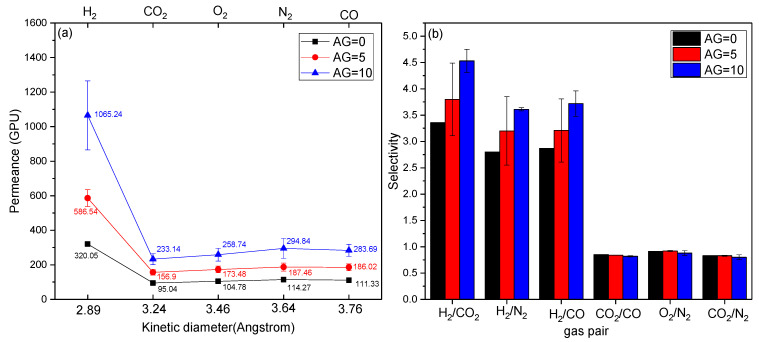
Effect of the air gap distance on (**a**) gas permeance and (**b**) selectivity of the as-obtained MMHFMs (used filler size: 1000 nm; flow rate ratio: 5.14).

The pure gas permeation measurements were carried out to evaluate the gas separation performance of MMHFMs with varying air gap distances, and the results are presented in Figure 4. The gas permeance of all MMCHFMs exhibited the following order: H_2_ (2 Daltons) >N_2_ (28 Daltons) > CO (28 Daltons) > O_2_ (32 Daltons) > CO_2_ (44 Daltons). The value in the bracket refers to the corresponding molecular weight for tested gas. Noticeably, the gas permeance value did not correlate with the kinetic diameter of the gas molecules. However, there was a trend between the gas permeance value and the reciprocal of the square root of the molecular weight. The typical characteristic displayed by Knudsen diffusion was that the gas permeance was inversely proportional to the molecular weight of the penetrated species [3]. This means the gas transport mechanisms of all MMCHFMs with different air gap distances were dominated by Knudsen diffusion, instead of molecular sieving behavior. As a common selective diffusion process [20], Knudsen diffusion has a distinctive potential for the resultant fiber in terms of commercialization, due to high permeance and adequate selectivity over gas pairs [12]. For instance, the AG10-R5.14-1000 nm fiber exhibited hydrogen permeance of 1065.24 GPU and selectivity values of hydrogen over carbon dioxide and hydrogen over monoxide of 4.53 and 3.72, respectively, which approached the ideal Knudsen separation factors (4.69 and 3.74). With an increase of the air gap distance, the hydrogen permeance, as well as separation factors for H_2_/CO_2_, H_2_/N_2_, and H_2_/CO, were enhanced, which is the combined result of the reduction in the length of the outer finger-like macrovoids and membrane thickness. This finding was confirmed by the observations from SEM analysis (Figure 2), and was in agreement with previous works [21,22]. In addition, it is noteworthy that the permeance of hydrogen significantly prevailed over other penetrates, mirroring the synergism effect caused by other mechanisms in parallel with Knudsen diffusion. The boosted H_2_ permeance could be attributed to its most narrow critical dimension, compared with that of other tested gases.

#### 3.3.2. Effect of the Flow Rate Ratio

**Figure 5:**
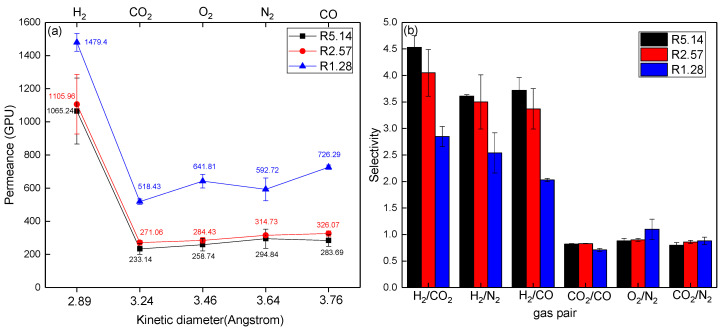
Effect of the flow rate ratio on (**a**) gas permeance and (**b**) selectivity of the obtained MMHFMs (used filler size: 1000 nm; air gap distance: 10 cm).

To investigate the influence of the flow rate ratio on the gas permeation property of the prepared fibers, the flow rate of the bore solution was adjusted from 5 to 20 mL/min. Meanwhile, the flow rate of the dope and the adopted air gap distance were kept constant as 25.7 mL/min and 10 cm, respectively. Subsequently, fibers spun with a flow rate ratio varying from 5.14 to 1.28 were fabricated and their permeation property for different tested gases was measured. Figure 5 depicts the plots of the permeation rate against the kinetic diameter of gas molecules, and the separation factors versus different gas couples. It can be seen that the gas molecule transport through the obtained MMHFMs were sequenced in the order of molecular mass. This indicates that the predominant transport behavior through the fibers was still Knudsen diffusion. When the flow rate ratio decreased from 5.14 to 1.28, the hydrogen permeance was increased almost 1.5 times, whereas the selectivity of H_2_/CO_2_ and H_2_/CO dropped to 2.85 and 2.03, respectively; a significant reduction of 37% and 45%, respectively. This can be attributed to the loosely packed membrane structure, consisting of a number of finger-like voids. The lower flow rate ratio caused much non-solvent liquid to flow into the lumen side of the fiber, promoting the non-solvent diffusion rate and inducing prompt phase inversion. As per the work of Zhu et al. [17], instantaneous phase inversion facilitates the formation of more and larger finger-like voids, resulting in an inferior gas separation performance, which was consistent with the trend observed in this section.

#### 3.3.3. Effect of Filler Size

**Figure 6:**
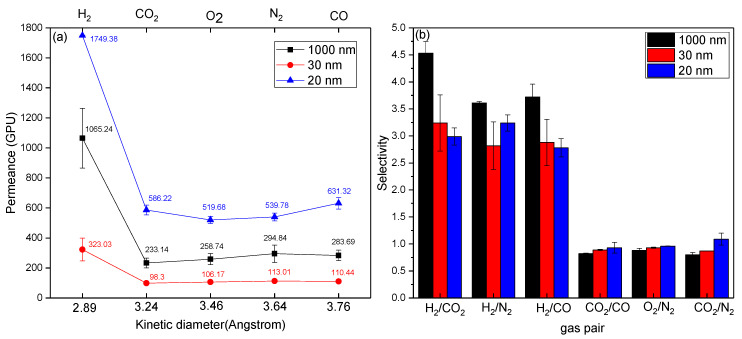
Effect of filler size on (**a**) gas permeance and (**b**) selectivity of the obtained MMHFMs (flow rate ratio: 5.14; air gap distance: 10 cm).

In this section, we examined the effect of particle size of the filler on the gas transport properties of the obtained fibers by incorporating different sizes of commercial alumina powders, of 1000 nm, 30 nm, and 20 nm, at a fixed loading weight of 6 wt. %. As shown in Figure 6, the hydrogen permeance of the fibers produced with 20 nm fillers reached 1749.38 GPU with H_2_/CO_2_ of 2.99. In comparison with 1000 nm and 30 nm, its permeance was significantly higher, while its separation capacity was inferior. This suggested the presence of non-selective pores for AG10-R5.14-20nm fibers. It is well known that smaller nanoparticles are prone to forming aggregates, because of their higher surface energy [23]. An unfavorable filler dispersion in the polymer matrix would result in defects between the inorganic phase and organic phase, and lower the gas separation performance. Moreover, it is clear that AG10-R5.14-1000 nm exhibited the highest improvement in hydrogen permeance and separation capacity for hydrogen enrichment, compared to other fibers with smaller particle sizes in the filler. This was due to the good compatibility between 1000 nm alumina fillers and the PEI matrix, which was corroborated with the EDX mapping analysis. It would be noteworthy to emphasize that the greater particle size enables the derived MMHFM to obtain the enhancement in permeance and H_2_/CO_2_ selectivity, which was attributed to better filler homogeneity and pore accessibility for penetrates. On the other hand, the lower nano-size of fillers may would cause either incomplete sealing of interfacial defects or partial pore blockage of fillers. For example, AG10-R5.14-30 nm MMHFM suffered a compromise in gas permeance. That is because of the partial clogging of filler occupied by polymer chains [24].

### 3.4. The Potential of Hydrogen Recovery from Either CO_2_ or Hydrocarbons

**Figure 7:**
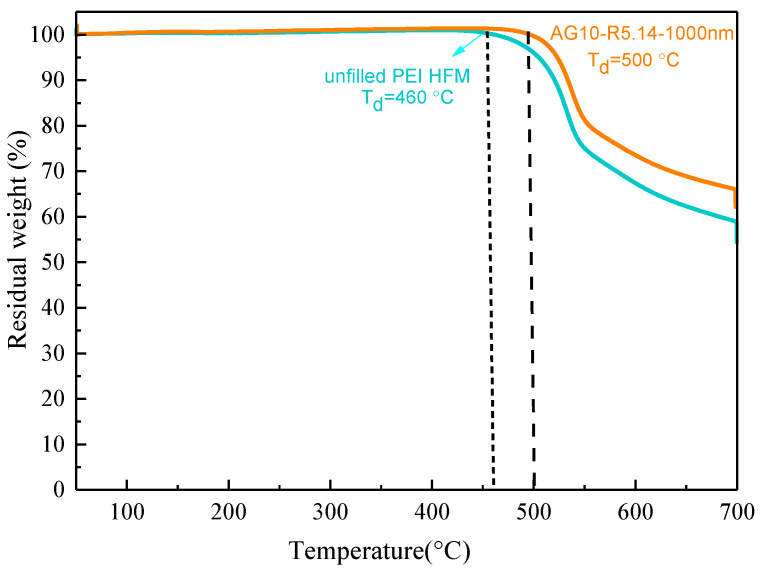
TGA profile of hollow fiber membrane spun at the same parameters with/without the incorporation of alumina filler.

In order to assess the performance of the MMHFMs synthesized in this work, the optimal fiber identified by the preceding discussions (AG10-R5.14-1000 nm) was compared with other membranes reported by previous studies. N. Muradov [25] pointed out that hydrogen production typically reaches up to 1 million m^3^ per day in the plant via the steam methane reformation process, with CO_2_ emissions of 0.4 million m^3^ per day. A membrane with high permeance and adequate selectivity is needed to satisfy the requirements of reforming gas mixture. In addition, recovery of hydrocarbons from gas streams is of prime importance for the chemical process industry from an economic point of view [12]. In the case of dehydrogenation of ethane (C_2_H_6_ → C_2_H_4_ + H_2_), where ethane was catalytically converted into ethylene and hydrogen with a Pt-based catalyst, this reaction not only yields ethylene, a crucial feedstock for a variety of petrochemicals (i.e., polymers and intermediates) [26], but also produces hydrogen at the same time. However, the conversion rate of alkane dehydrogenation is inhibited by thermodynamic equilibrium, so elevating the operation temperature or lowering the partial pressure of the product is considered the appropriate method to overcome this and improve reaction efficiency, mainly thanks to its endothermic feature [27]. Accordingly, the H_2_-selective membrane is desirable for this application, to effectively extract hydrogen from gas mixtures containing heavier hydrocarbons. The permeation properties of the screened MMHFM (AG10-R5.14-1000 nm) was also examined by the permeation measurement for common hydrocarbons (ethane and propane) to evaluate its potential for emerging applications. Table 4 summarizes the separation performance of as-obtained HFM with/without the presence of fillers as well as the other asymmetric membrane [28] and conventionally used polymeric membranes for alkane separation [29,30,31,32,33]. However, some papers did not provide the complete information regarding thickness of selective layer, which results in unsuccessful conversion between GPU and Barrer. By considering showcasing the potential of as-synthesized MMHFM for hydrogen purification from carbon dioxide or lighter hydrocarbons, hydrogen permeance and permeability were listed together. In comparison with the unfilled HFM and previously published asymmetric membrane [28], the superiorities of both hydrogen permeance and selectivity of H_2_/CO_2_ were obviously observed in the optimal MMHFM. This improvement could be attributed to the combination of the progress made by mixed matrix type membrane and the favorable synergism caused by the fillers and PEI matrix. Furthermore, compared with widely used polymeric membranes, the optimal MMHFM in the current study exhibited impressive H_2_ permeance and sufficient alkane separation factors. Based on this comparative result, the resulting MMHFMs could be used for the separation of hydrogen from either carbon dioxide or hydrocarbons. Moreover, it is interesting to note that the selectivity toward H_2_/C_3_H_8_ was lower than that for H_2_/C_2_H_6_. In general, permeance decreases with an increase in the kinetic diameter of penetrants. Thanks to the bigger kinetic diameter of propane (4.3 angstrom) in comparison with that of ethane (3.85 angstrom), H_2_/C_3_H_8_ selectivity is assumed to top that of H_2_/C_2_H_6_. This intriguing phenomenon was attributed to the stronger condensability of propane [34], resulting in an inferior separation factor. This finding was also observed in other literature [29]. It should be noted that the reaction temperature of ethane dehydrogenation is commonly high and a broad temperature range (300–1000 °C) based on the selected production process including steam cracking process (above than 1000 °C), catalytic dehydrogenation (550–700 °C), and oxidative dehydrogenation (350–600 °C) [35,36]. To confirm the adaptability of resultant MMHFM in this application, its thermal stability was examined by TGA analysis. Based on the profile (Figure 7), the decomposition temperature (Td) of unfilled PEI HFM was approximately at 460 °C, whereas that of resultant MMHFM reached up to 500 °C. Considering the thermal stability result, we inferred that optimal MMHFM is more suitable for serving as the role of separation barrier after the dehydrogenation instead of ongoing reaction. Furthermore, the temperature for aromatic/aliphatic separation by commonly used polymeric membrane lies in the range of 25–70 °C, as pointed out by Gugliuzza and Basile [37], which mirrors the breakthrough that was achieved by this work in terms of thermal stability during separation.

## 4. Conclusions

In this study, a fixed number of commercial alumina nanoparticles were successfully incorporated as fillers into a polyetherimide (PEI) matrix to prepare a mixed matrix hollow fiber membrane (MMHFM). Due to the presence of hydrogen bond interactions between the hydroxyl groups of alumina powder and the C-H segment in the PEI chain, good compatibility between both phases was confirmed by EDX-mapping analysis. Additionally, the effect of the spinning parameters, including the air gap distance and flow rate ratio, on the morphologies and gas transport properties of the prepared MMHFMs was systematically investigated by SEM and single gas permeation measurement, respectively. The results showed both spinning parameters had a positive influence on the gas separation performance of the MMHFMs. The improved performance in hydrogen permeance and selectivity was obtained by setting the spinning parameters as follows: air gap distance of 10 cm and flow rate ratio of 5.14. This can be attributed to the reduction in the region occupied by finger-like macrovoids, and membrane thickness. Subsequently, the effect of filler size was studied by adding three sizes of commercial alumina particles in the range of 20–1000 nm into the polymeric phase. The permeation results indicated a smaller filler would cause incompatibility in the interface due to its high surface energy, thus lowering the gas separation capacity. Finally, compared with other membranes reported by previous studies, the optimal MMHFM (AG10-R5.14-1000 nm) displayed an excellent hydrogen permeance of 1065.24 GPU, and sufficient selectivity for the H_2_/CO_2_, H_2_/C_2_H_6_, and H_2_/C_3_H_8_ gas pairs of 4.53, 5.77, and 5.39, respectively, which reflected its significant potential for the purification of hydrogen and/or recovery of lower hydrocarbons.

## Figures and Tables

**Table 1:** Summarized spinning parameters.

Condition	Value
**Composition of spinning mixture (Al_2_O_3_:NMP:PEI)**	6:75:25 (weight ratio)
**Bore liquid**	D.I. water
**External coagulant**	tap water
**Dope flow rate (mL/min)**	25.7
**Air gap (cm)**	0, 5, 10
**Bore liquid flow rate (mL/min)**	5, 10, 20

**Table 2:** The effect of the air gap distance on the membrane thickness.

Sample Code	Membrane Thickness (μm)	Length of Outer Finger-Like Structure (μm)
**AG0-R5.14-1000 nm**	786.4	250.5
**AG5-R5.14-1000 nm**	443.7	173.0
**AG10-R5.14-1000 nm**	393.2	153.9

**Table 3:** The effect of the flow rate ratio on the membrane thickness.

Sample Code	Membrane Thickness (μm)	Length of Inner Finger-Like Structure (μm)
**AG10-R1.28-1000 nm**	711.1	366.2
**AG10-R2.57-1000 nm**	548.2	294.3
**AG10-R5.14-1000 nm**	393.2	224.7

**Table 4:** Comprehensive comparison with other asymmetric membranes and commonly used membranes for alkane separation.

Membrane	Hydrogen Permeance (GPU)	Thickness of Selective Layer (μm)	Hydrogen Permeability (Barrer) *	Selectivity			Ref.
				H_2_/CO_2_	H_2_/C_2_H_6_	H_2_/C_3_H_8_	
**Unfilled PEI**	26.26	-	-	3.52	-	-	This work
**PEI/Al_2_O_3_**	1065.24	-	-	4.53	5.77	5.39	
**PI/PSf**	348	-	-	4.4	-	-	[28]
**Poly(4-methyl-2-pentyne)**	94–141	40–60	$5.64\times 10^{3}$	-	1.51	1.35	[29]
**Poly(6-methyl-2-heptyne)**	12.5–18.75	40–60	$0.75\times 10^{3}$	-	2.67	-	[29]
**PIM-1**	58.6	50	$2.93\times 10^{3}$	-	7.87	-	[30]
**Poly(4-methyl-2-pentyne)**	-	-	$5.80\times 10^{3}$	-	1.56	1.23	[31]
**Poly(trimethylsiloxy)silyl)-tricyclonone**	-	-	$0.69\times 10^{3}$	-	-	1.32	[32]
**Poly(2-hexyne)**	2.66–4	40–60	$0.16\times 10^{3}$	-	10	4.57	[33]

* 1 Barrer = GPU×selective layer thickness (μm)= 10^−10^ cm^3^ (STP) cm cm^−2^ s^−1^ cmHg^−1^. - refers to the information which was not provided in the reference.

## References

[1] Lei L., Pan F., Lindbråthen A., Zhang X., Hillestad M., Nie Y., Bai L., He X., Guiver M.D. (2021). Carbon hollow fiber membranes for a molecular sieve with precise-cutoff ultramicropores for superior hydrogen separation. Nat. Commun..

[2] Du Z., Liu C., Zhai J., Guo X., Xiong Y., Su W., He G. (2021). A review of hydrogen purification technologies for fuel cell vehicles. Catalysts.

[3] Lin Y.-T., Zhuang G.-L., Wey M.-Y., Tseng H.-H. (2020). The viable fabrication of gas separation membrane used by reclaimed rubber from waste tires. Polymers.

[4] Schmeda-Lopez D.R., Smart S., Meulenberg W.A., Diniz da Costa J.C. (2017). Mixed matrix carbon stainless steel (mmcss) hollow fibres for gas separation. Sep. Purif. Technol..

[5] Kumakiri I., Tamura K., Sasaki Y., Tanaka K., Kita H. (2018). Influence of iron additive on the hydrogen separation properties of carbon molecular sieve membranes. Ind. Eng. Chem. Res..

[6] Widiastuti N., Gunawan T., Fansuri H., Salleh W.N.W., Ismail A.F., Sazali N. (2020). P84/zcc hollow fiber mixed matrix membrane with pdms coating to enhance air separation performance. Membranes.

[7] Roslan R.A., Lau W.J., Lai G.S., Zulhairun A.K., Yeong Y.F., Ismail A.F., Matsuura T. (2020). Impacts of multilayer hybrid coating on psf hollow fiber membrane for enhanced gas separation. Membranes.

[8] Ahmadi M., Janakiram S., Dai Z., Ansaloni L., Deng L. (2018). Performance of mixed matrix membranes containing porous two-dimensional (2d) and three-dimensional (3d) fillers for CO_2_ separation: A review. Membranes.

[9] Laghaei M., Sadeghi M., Ghalei B., Shahrooz M. (2016). The role of compatibility between polymeric matrix and silane coupling agents on the performance of mixed matrix membranes: Polyethersulfone/mcm-41. J. Membr. Sci..

[10] Xing P., Robertson G., Guiver M., Mikhailenko S., Wang K., Kaliaguine S. (2004). Synthesis and characterization of sulfonated poly(ether ether ketone) for proton exchange membranes. J. Membr. Sci..

[11] Dai Y., Ruan X., Yan Z., Yang K., Yu M., Li H., Zhao W., He G. (2016). Imidazole functionalized graphene oxide/pebax mixed matrix membranes for efficient CO_2_ capture. Sep. Purif. Technol..

[12] Wey M.-Y., Chen H.-H., Lin Y.-T., Tseng H.-H. (2020). Thin carbon hollow fiber membrane with knudsen diffusion for hydrogen/alkane separation: Effects of hollow fiber module design and gas flow mode. Int. J. Hydrogen Energy.

[13] Awanis Hashim N., Liu F., Moghareh Abed M.R., Li K. (2012). Chemistry in spinning solutions: Surface modification of pvdf membranes during phase inversion. J. Membr. Sci..

[14] Raharjo Y., Wafiroh S., Nayla M., Yuliana V., Fahmi M.Z. (2017). Primary study of cellulose acetate hollow fiber as a green membrane applied to hemodialysis. J. Chem. Technol. Metall..

[15] Hamid N., Ismail A., Matsuura T., Zularisam A., Lau W., Yuliwati E., Abdullah M. (2011). Morphological and separation performance study of polysulfone/titanium dioxide (psf/tio_2_) ultrafiltration membranes for humic acid removal. Desalination.

[16] Khan I.U., Othman M.H.D., Ismail A., Matsuura T., Hashim H., Nordin N.A.H.M., Rahman M.A., Jaafar J., Jilani A. (2018). Status and improvement of dual-layer hollow fiber membranes via co-extrusion process for gas separation: A review. J. Nat. Gas Sci. Eng..

[17] Zhu H., Jie X., Wang L., Kang G., Liu D., Cao Y. (2018). Enhanced gas separation performance of mixed matrix hollow fiber membranes containing post-functionalized s-mil-53. J. Energy Chem..

[18] Praneeth K., James T., Sridhar S. (2014). Design of novel ultrafiltration systems based on robust polyphenylsulfone hollow fiber membranes for treatment of contaminated surface water. Chem. Eng. J..

[19] Li G., Kujawski W., Knozowska K., Kujawa J. (2021). The effects of pei hollow fiber substrate characteristics on pdms/pei hollow fiber membranes for co_2_/n_2_ separation. Membranes.

[20] Nagy E. (2018). Basic Equations of Mass Transport through a Membrane Layer.

[21] Hasbullah H., Kumbharkar S., Ismail A., Li K. (2011). Preparation of polyaniline asymmetric hollow fiber membranes and investigation towards gas separation performance. J. Membr. Sci..

[22] Zulhairun A., Ng B., Ismail A., Murali R.S., Abdullah M. (2014). Production of mixed matrix hollow fiber membrane for co_2_/ch_4_ separation. Sep. Purif. Technol..

[23] Wang F., Zheng T., Xiong R., Wang P., Ma J. (2019). Strong improvement of reverse osmosis polyamide membrane performance by addition of zif-8 nanoparticles: Effect of particle size and dispersion in selective layer. Chemosphere.

[24] Dong G., Li H., Chen V. (2013). Challenges and opportunities for mixed-matrix membranes for gas separation. J. Mater. Chem. A.

[25] Muradov N., Subramani V., Basile A., Veziroğlu T.N. (2015). 17—low-carbon production of hydrogen from fossil fuels. Compendium of Hydrogen Energy.

[26] Benali M., Aydin B. (2010). Ethane/ethylene and propane/propylene separation in hybrid membrane distillation systems: Optimization and economic analysis. Sep. Purif. Technol..

[27] Ahn S.-J., Yun G.-N., Takagaki A., Kikuchi R., Oyama S.T. (2018). Effects of pressure, contact time, permeance, and selectivity in membrane reactors: The case of the dehydrogenation of ethane. Sep. Purif. Technol..

[28] Hamid M.A.A., Chung Y.T., Rohani R., Junaidi M.U.M. (2019). Miscible-blend polysulfone/polyimide membrane for hydrogen purification from palm oil mill effluent fermentation. Sep. Purif. Technol..

[29] Pinnau I., He Z., Morisato A. (2004). Synthesis and gas permeation properties of poly(dialkylacetylenes) containing isopropyl-terminated side-chains. J. Membr. Sci..

[30] Li P., Chung T., Paul D. (2013). Gas sorption and permeation in pim-1. J. Membr. Sci..

[31] Morisato A., Pinnau I. (1996). Synthesis and gas permeation properties of poly(4-methyl-2-pentyne). J. Membr. Sci..

[32] Bermeshev M.V., Syromolotov A.V., Starannikova L.E., Gringolts M.L., Lakhtin V.G., Yampolskii Y.P., Finkelshtein E.S. (2013). Glassy polynorbornenes with si–o–si containing side groups. Novel materials for hydrocarbon membrane separation. Macromolecules.

[33] Pinnau I., Morisato A., He Z. (2004). Influence of side-chain length on the gas permeation properties of poly (2-alkylacetylenes). Macromolecules.

[34] Mukaddam M., Litwiller E., Pinnau I. (2016). Gas sorption, diffusion, and permeation in nafion. Macromolecules.

[35] Fairuzov D., Gerzeliev I., Maximov A., Naranov E. (2021). Catalytic dehydrogenation of ethane: A mini review of recent advances and perspective of chemical looping technology. Catalysts.

[36] Gärtner C.A., van Veen A.C., Lercher J.A. (2013). Oxidative dehydrogenation of ethane: Common principles and mechanistic aspects. ChemCatChem.

[37] Gugliuzza A., Iulianelli A., Basile A., Basile A., Nunes S.P. (2011). 10—membranes for hydrocarbon fuel processing and separation. Advanced Membrane Science and Technology for Sustainable Energy and Environmental Applications.

